# Editorial

**Published:** 1986-12

**Authors:** 


### Editorial

### THE FIRST YEAR OF THE NEW JOURNAL

This number completes Volume 101 of the Bristol Medico-Chirurgical Journal and the first volume of Bristol Medicine, the fruit of cooperation between Hermiston Publications and the Bristol Medico-Chirurgical Society. As a result the Journal has appeared bimonthly instead of quarterly and has been distributed free to all doctors in the West of England instead of on payment to its subscribers. This change has been made possible by the sponsorship arrangements which Dr Ian McKee and Hermiston publications have been able to make with advertising drug companies for the journals which they publish – Edinburgh, Glasgow, Scottish, Manchester and London Medicine and our own Bristol Medicine. Most of the other journals have two sponsoring firms but our journal has been sponsored entirely by Bristol-Myers. We therefore wish to express again our most grateful thanks for this very generous support and we hope that the advertising that their products have received in our pages has been effective in making them known.

The sponsorship agreement has been for a 24 page number (yielding 18 pages of 'copy'). Formerly we would vary the number of pages to meet our needs and now a build-up of 'copy' makes it desirable to be able to increase the number of pages when necessary. The Bristol Medico-Chirurgical Society has agreed to contribute towards this extra cost for one year in the first instance, and the combined editorial board of the new journal wishes to express its gratitudes for this support.

There have unfortunately, but perhaps not surprisingly, been distribution problems. Some doctors have not received the journal and some have received more than one. The hospital distribution has been especially defective. The computer in Edinburgh into which the names were originally fed proved unable to cope with its task and has been replaced by a new one. We must ask for your help in telling us where we have failed (by writing to Hermiston Publications) and for your forbearance until we have got things right and after that for informing us of changes of address and so forth. We have also been slow to develop regular contribution covering the whole of our large territory. Taunton is supporting us, we have promises from Exeter and Plymouth, we would very much like to have regular correspondents from other parts of the West country. Volunteers please.

### MEDICINE AND THE TELEVISION

The Editor has to state that the pressure on space for his pages has inhibited him from editorialising since the initial number this year. Many will think that this is no bad thing. Perhaps however we should raise controversial issues from time to time. Mr Norman Tebbitt has fired a broadside at the BBC, the BBC has reacted strongly. Without question the BBC is a magnificent institution, enlarging the national awareness and enriching our lives immeasurably, it is worth the licence fee for David Attenborough alone. It is quite probably true, as we are so often told, that the BBC gives us the best broadcasting in the world, but it permeates our lives and influences our attitudes to everything and not always for the good. It can be infuriating and one can sympathise with Norman Tebbitt. Political bias is what he complains of and he may be right. It is more likely perhaps that the BBC is naturally 'agin the government' and the longer a government has been in power the more 'agin it' it becomes. Reporters are also critics and anyone who sticks his head above the parapet is asking to be shot at. Medicine is more and more 'news' and we are more and more targets. Many of us automatically switch off when medicine is 'on the box', they so often, we feel, somehow get it wrong. It is hard to see yourself as others see you. However we have to take what they throw at us and if necessary throw it back. Panorama will never be forgiven for its programme some years back on transplantation, in which it was implied that organs were removed before patients were incontrovertably dead, and they had to go to America for their supposed evidence. The result was to nearly dry up the suply of transplantable kidneys on which the lives of so many depend and it took a long time to recover. Some medical reporting is accurate and objective, and an Esther Rantzen 'That's Life' programme on Ben Hardwick, the child in need of a liver transplant remedied some of the damage 'Panorama' had inflicted. Nevertheless our inadequacies are always in the spotlight. The vast amount of hospital building that is going on all the time seems to be ignored, will the cameras show any interest in the beautiful new hospital at Weston-super-Mare? (See correspondents column.) Only, I suspect, if it is officially opened by a member of the Royal Family. If an old run-down hospital is to be closed, the anger of the local population at heartless Tory cuts is the theme. Of course doctors (or most of them) dislike the limelight and only wish to care for their patients in decent obscurity, any intrusion of cameras is distasteful. Unfortunately, we have to live with the modern obsession that everyone has the right to know everything, but we must make sure that the BBC, this twentieth century inquisition, gets it right and we can help them to do so. The new BBC science series 'Antenna' started well with a programme on the difficulties paediatricians have in deciding how far they should go in salvaging infants who, though viable, are irreparably brain damaged. How does ITVs medical reporting compare with the BBCs? This journal would be glad to hear their colleague's views on our Television coverage — objective and unbiased of course.

